# Non-innocent water in zeolite-based microporous liquids

**DOI:** 10.1039/d6cc03473j

**Published:** 2026-08-11

**Authors:** Deborah A. Brako-Amoafo, Nicole L. Kelly, Christopher J. Heard, Sharon E. Ashbrook, Russell E. Morris

**Affiliations:** a EaStCHEM School of Chemistry and Centre of Magnetic Resonance, North Haugh St Andrews KY16 9ST UK rem1@st-andrews.ac.uk; b Department of Physical and Macromolecular Chemistry, Faculty of Sciences, Charles University Hlavova 8 128 43 Prague 2 Czech Republic

## Abstract

Aqueous pure silica zeolite-based microporous liquids, also known as microporous water, show interesting properties based on their internal hydrophobic and external hydrophilic chemistry. Here we show, using ^17^O NMR spectroscopy that the water, in which the nanoparticle zeolites are dispersed, exchanges with the external surface of the pure silica material.

## Abstract 2

Solid-state ^17^O NMR spectroscopy shows that zeolite nanoparticles exchange oxygen atoms with water when dispersed in a microporous liquid.

## Graphical abstract

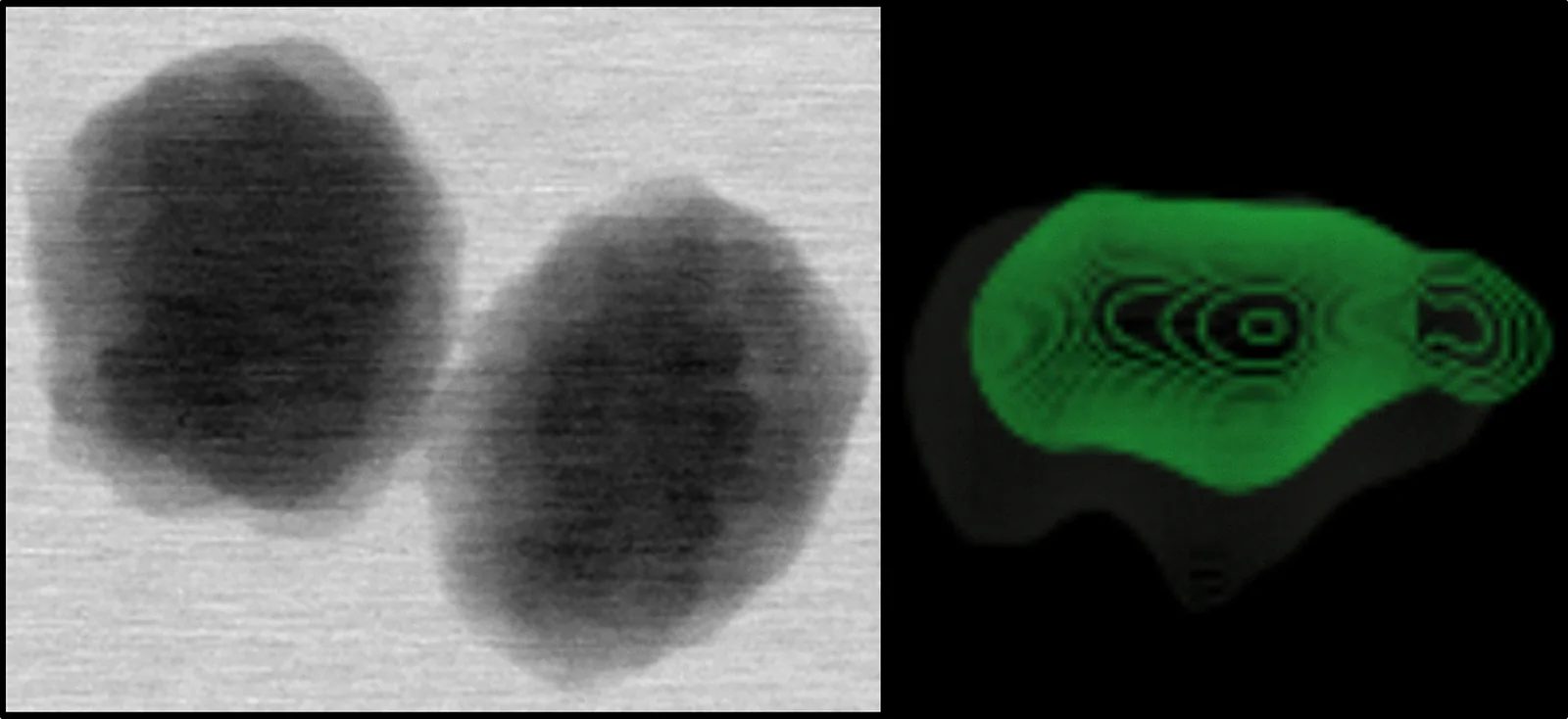

The concept of a microporous liquid was first proposed by James and co-workers in 2007.^1,2^ They are a class of novel materials that combine the properties of liquids and microporous solids, offering unique characteristics attractive for various applications, including selective gas transport, separations and catalysis.^3–5^ The key feature of a porous liquid is porosity at the molecular or nanoscale level, enabling selective absorption of gases, small molecules or ions while behaving as fluids that are compatible with familiar liquid-handling infrastructure and with liquid-based systems including biology. The development of microporous liquids represents an exciting frontier in materials science, bridging the gap between liquids and solid-state porous materials. One type of microporous liquid (so-called type 3)^1,2^ is defined as the stable dispersion of nanoparticles (NPs) of a microporous solid in a carrier solvent to form a stable colloid. This remains a hugely under-developed concept because creating porous NPs that are compatible with a carrier solvent but exclude that solvent from their interior pores is non-trivial.

In a recent paper in Nature, Mason and coworkers have developed the concept of zeolite-based “microporous water”, which is comprised of stable dispersions of a microporous zeolite NPs in water.^6^ Rather than relying on size mismatch using unusual carrier solvents – an earlier approach^7–11^ – Mason applied NPs that possess water-compatible (hydrophilic) exteriors but water-rejecting (hydrophobic) interiors (Fig. 1). This new class of porous liquid has some outstanding properties as oxygen storage and transport media for use in catalysis and physiological gas transport. Careful measurement of the overall density of the microporous liquid suggested that no (or at least very little) water intrudes into the dispersed nanoparticles,^6^ although this does depend on the level of defects present in the material.^12^ However, here we show that despite this, the role of water is not simply as an innocent solvent in which the zeolites are dispersed, but that the oxygen atoms from the water exchange with those in the zeolite nanoparticles. We prove this using solid-state ^17^O MAS NMR spectroscopy of the NPs after they have been separated from the liquid.

**Fig. 1:**
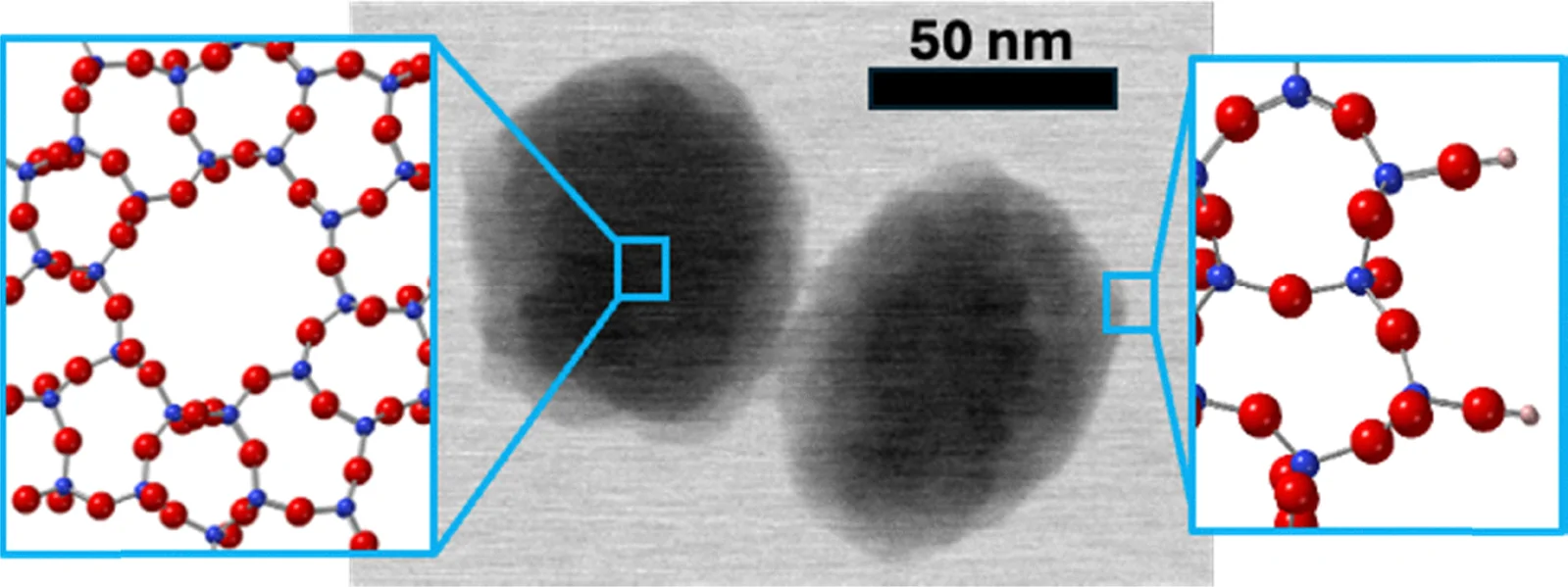
The chemical principles on which microporous water is based. The external surface of zeolite nanoparticles (right) comprises hydrophilic Si–OH groups, while the interior of the particle (left) consists of hydrophobic Si–O–Si. The central image is from TEM. Key: Si = blue; O = red; H = pink.

Zeolite–water interactions are a topic of keen interest as recent studies have shown that the bonds in zeolites are labile at room temperature when the materials are in contact with liquid water.^13–16^ A particular surprise was the identification that purely siliceous zeolites with large crystallites (>1 µm) exchange oxygen atoms quickly at room temperature when in contact with liquid water – this changes our view of what even supposedly hydrophobic zeolites look like under such mild conditions.^17^ We show here that the NPs behave differently to large crystals by being relatively more susceptible to exchange at their surface, which can be related to the nanoparticulate nature of the zeolites in the microporous liquids. The results show that water is not simply an innocent liquid in which the zeolite is dispersed but is reacting reversibly with the nanoparticles.

Nanosized pure silica zeolite MFI (also known as silicalite-1) was synthesised using a modified version of the published synthesis procedure^18,19^ and the structure confirmed using powder X-ray diffraction (Fig. S1) The as-made nanosized MFI was calcined at 600 °C for six hours to remove the tetrapropylammonium hydroxide (TPAOH) organic structure directing agent (OSDA), and then characterised using solid-state NMR spectroscopy (Fig. 2 and 3), scanning electron microscopy (SEM, Fig. S5), transmission electron microscopy (TEM, Fig. S6–S8) and dynamic light scattering (DLS). All the synthesis and characterisation data can be found in the (SI). DLS showed that the product had an average hydrodynamic radius of ∼60–65 nm when dispersed in water (Table S1), and the SEM imaging (Fig. S5) and TEM imaging (Fig. S6–S8) showed almost spherical crystallites after the nanoparticles had been recovered and dried.

**Fig. 2:**
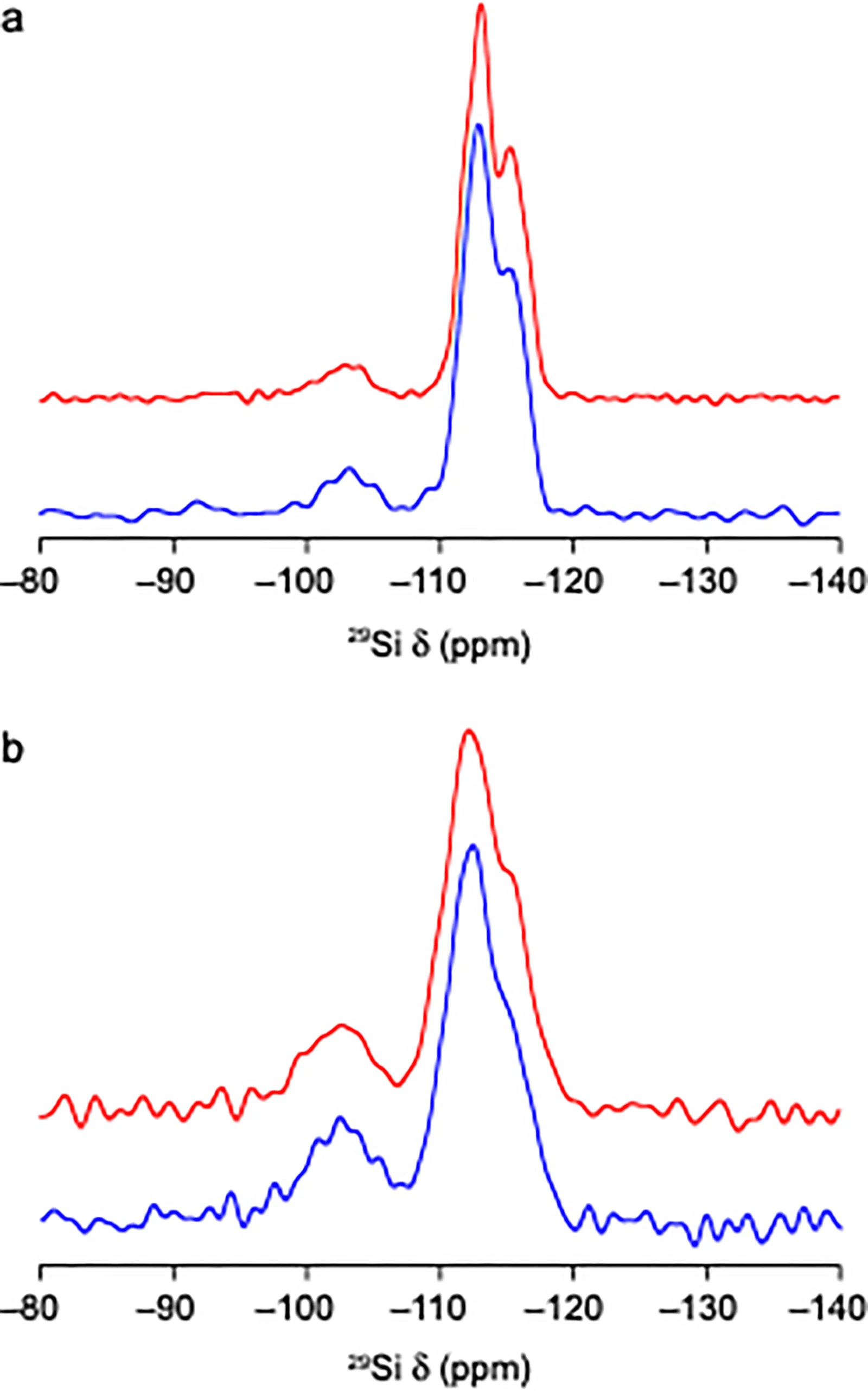
^29^Si (14.1 T, 10 kHz) MAS NMR spectra of (a) calcined and (b) as-made MFI nanoparticles before (blue) and after (red) dispersion in 40% ^17^O-enriched water for six days.

The ^29^Si magic angle spinning (MAS) NMR measurements of both as made and calcined nanoparticles shown in Fig. 2 showed clear sets of signals, each resulting from multiple crystallographic sites, at −113 and −104 ppm, which can be attributed to Q^4^ (Si(OSi)_4_) and Q^3^ (Si(OSi)_3_(OH))-type silicon, respectively. Additional ^1^H–^29^Si cross-polarisation (CP) MAS NMR spectra showed relative enhancements in the intensity of signal at −104 ppm confirming that the Q^3^ silicon is in closer proximity to protons than the Q^4^ silicon sites, as expected. It also revealed the presence of a very small number of Q^2^ sites (Fig. S3); the signal from these species is not visible in the single pulse ^29^Si MAS NMR spectra at this signal-to-noise ratio. Measurement of the relative intensities of the signals in the single-pulse NMR spectra indicates that the Q^3^ sites make up ∼10% of the total intensity. This is rather more than seen in many large crystal samples of MFI but can be explained by the increased amount of surface silanol species relative to the bulk in nanoparticles compared to microcrystals. However, it should be remembered that zeolites also contain several different types of defects in their bulk structure, which also contribute to the Q^3^ signal.^12,20,21^

Samples of ∼50 mg of both calcined and as-made nanosized MFI were dispersed in 0.5 mL 40 wt% ^17^O-enriched water at room temperature for six days. Subsequently, the nanoparticles were recovered from the liquid using centrifugation and then dried in an oven overnight at 80 °C, which removes any weakly adsorbed water from the sample (Fig. S2). After contact with ^17^O-enriched water the samples were characterised using ^29^Si and ^17^O solid-state NMR spectroscopy to observe any changes in the local structure of the zeolite. ^29^Si MAS NMR spectra (Fig. 2) showed no major changes in the spectra after exposure to water for six days. The changes in both the Q^3^ and Q^4^ signals are very small and show no major degradation of the crystalline nature of the materials. This is supported by PXRD, SEM and TEM (as shown in the SI), all of which showed no significant changes after contacting with ^17^O-enriched water.

The low natural abundance of ^17^O (0.038%) means that in the calcined samples no ^17^O NMR spectra can be acquired on any reasonable timescale (Fig. S4). However, after contact with 40% ^17^O-enriched water as described above there is clear evidence of signals in both the ^17^O MAS and the MQMAS NMR spectra (Fig. 3) for the presence of Si–^17^O–Si species in the sample. See the SI for more detail on the MQMAS experiment. As seen in previous work on silicate,^17^ aluminosilicate^13,16^ and germanosilicate^22–25^ zeolites, these oxygen nuclei have *δ* iso of 39–45 ppm and *C*_Q_ and/or *P*_Q_ values of 5–5.7 MHz. Any dynamic water adsorbed into the pores would have *δ* iso of 0 ppm and *C*_Q_ = 0 MHz, and so are not seen in MQMAS NMR spectra. Therefore, the ^17^O MAS and MQMAS NMR spectra indicate that the oxygen atoms in the zeolite framework are exchanging with those from the water. This means that in a sample of microporous water the liquid is not an innocent chemical species in which the porous solid nanoparticles are simply dispersed but is actively interacting and exchanging with the zeolite material. It might also be expected to see signals from the Si–^17^OH species that will be found on the surface of the particles; however, these signals are challenging to see in MQMAS spectra (or indeed cross polarisation spectra) unless extremely high decoupling powers are used owing to their dynamics and rapid relaxation.^23^

The overall isotope exchange reaction is reversible in nature as no degradation of crystallinity is seen with any of the characterisation techniques used. The absolute ^17^O enrichment level was measured at ∼7% by comparing the intensity of the NMR spectroscopy signal with a standard sample of ^17^O-enriched alumina, using a method that has been previously published.^17^

The elegant density studies completed by Erdosy *et al.*^6^ showed that in a microporous liquid of this kind there is very little water ingress into the nanoparticles – a result ascribed to the high hydrophobicity of the interior of a pure silica zeolite such as MFI. Recent work on pure silica zeolite microcrystals showed that despite this hydrophobicity, exchange of oxygen atoms within the bulk of the crystals is facile even for relatively low-defect zeolite samples, with considerable exchange (up to 40–50%) of ^17^O or ^18^O atoms measured using solid-state NMR spectroscopy and neutron diffraction for AFI, CHA, FER and MFI frameworks.^17^ These results seem, at first, to be at odds with each other. However, the dynamic nature of the water-zeolite interaction means that even when there is only a small amount of water within the pores of the zeolite then the isotope exchange reaction proceeds relatively easily. Nevertheless, one question remains unanswered – does the size of the crystallites matter?

Given the external surface area of the crystallite becomes more important relative to the bulk as crystal size decreases, it can be surmised that this may have an influence on any isotope exchange process. It is very likely that clustering of water will be nucleated at silanol sites in zeolites, which has been shown computationally to be likely important in the overall exchange mechanism.^13^ To probe whether the increased number of surface silanols in a nanoparticle is an important factor we completed the same microporous water experiment but using the as-made MFI material. In these nanoparticles the pores of the zeolite still contain the tetrapropylammonium OSDA cation, which effectively blocks access to the interior of the nanoparticle; *i.e.*, the as-made material is non-porous. This allows us to probe whether the isotope exchange process takes place at the surface of the nanoparticles or whether a measurable amount takes place at the internal surface of the solids, which is only possible for the calcined, porous zeolite.

^17^O MAS NMR spectra of the as-made nanoparticles after forming microporous water using 40% ^17^O-enriched liquid, showed a very similar result to that of the calcined sample (Fig. 3), with clear evidence of Si–^17^O–Si units being formed in the nanoparticles. Once differences in the number of transients averaged and sample mass had been accounted for, very similar signal intensities were observed for the two samples. Given that the presence of the OSDA in the pores precludes access to the internal surface of the zeolite it is reasonable to suggest that it is the external surface of the zeolite that is primarily involved in the isotopic exchange for this system. This is somewhat different to the case for micron-sized crystals where exchange occurs at more than just the surface.^17^ However, the surface area: bulk volume ratio changes by more than 2 orders of magnitude between nanoparticles of 60 nm diameter and crystals of 1 µm diameter. It is therefore not surprising that surface effects are more important in the nanoparticles. Indeed, it is well established that nanoparticles of zeolites and other porous materials such as MOFs have very different properties to their microcrystal equivalents.^26^ The observation of isotope exchange is vital in determining the behaviour of microporous liquids, as unlike the previous theories of this type 3 microporous water it is now clear that the liquid is not simply an innocent phase in which the porous solids are dispersed but is a potentially reactive matrix. This reactivity could have an impact on aspects such as the long-term stability of a microporous water dispersion and so it is important to understand the interaction of the liquid matrix with the porous solid nanoparticles if we are to develop, for example, applications of these fascinating systems further.

**Fig. 3:**
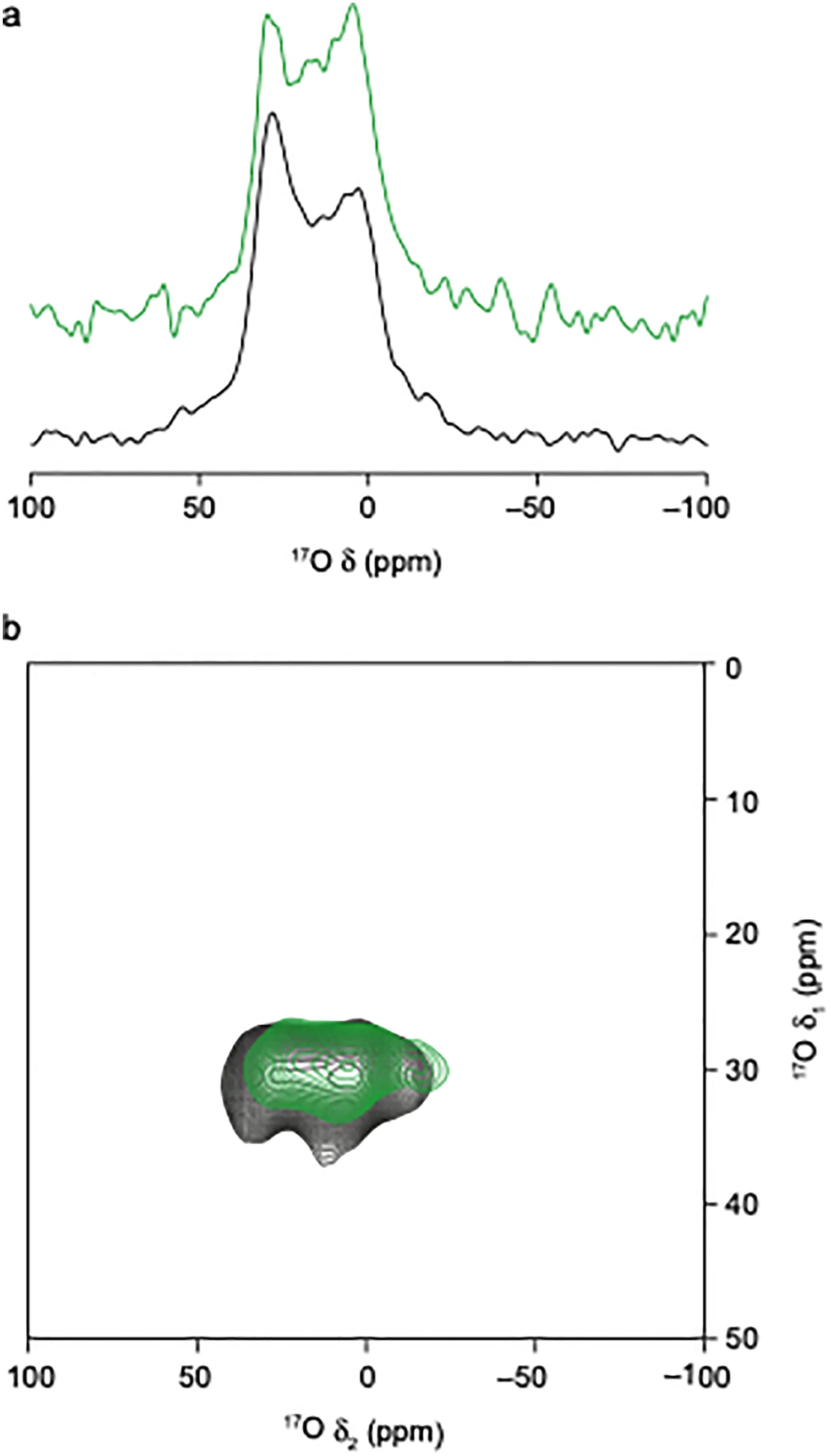
^17^O (14.1 T, 20 kHz) (a) MAS and (b) MQMAS NMR of as-made MFI (black) and calcined MFI (green) showing the similar signal from the Si–^17^O–Si units after dispersing the nanoparticles in 40% ^17^O-enriched water for six days.

## Conflicts of interest

There are no conflicts to declare.

## Supplementary Material

CC-062-D6CC03473J-s001

## Data Availability

The data supporting this article have been included as part of the supplementary information (SI). Supplementary information is available. See DOI: https://doi.org/10.1039/d6cc03473j. The raw data (NMR spectroscopy and XRD) supporting this publication can also be accessed at https://doi.org/10.17630/22ddcd41-074e-4523-9877-e3e926f5a53b.
