# Supplementary material for: Non-innocent water in zeolite-based microporous liquids
Source: Chem Commun (Camb). 2026 Aug 11;62(70):17497–500. doi: 10.1039/d6cc03473j (PMC13479698; doi:10.1039/d6cc03473j)
Supplement: Supplementary file 1 [file CC-062-D6CC03473J-s001.pdf]

Supplementary Information (SI) for Chemical Communications.
This journal is © The Royal Society of Chemistry 2026

Supplementary Information

Non-innocent water in zeolite-based microporous liquids

Deborah A. Brako-Amoafo[a,b], Nicole L. Kelly[a], Christopher J. Heard,[b] Sharon E. Ashbrook[a] and Russell E. Morris.[a]

a. *EaStCHEM School of Chemistry and Centre of Magnetic Resonance, North Haugh, St Andrews KY16 9ST, U.K.*
b. *Department of Physical andMacromolecular Chemistry, Faculty of Sciences, Charles University, Hlavova 8, 128 43 Prague 2, Czech Republic.*

S1 Synthesis and basic characterisation of nanosized siliceous MFI zeolite

The synthesis of siliceous MFI was based on the recipe published in the International Zeolite Association (https://www.iza-online.org) collection of verified syntheses book.[1, 2] The only modification was that centrifugation was completed at 14500 rpm for twenty minutes.

The powder X-ray diffraction (PXRD) pattern for the synthesised materials (Figure S1, Cu Kα radiation, PANanalytical Empyrean in Bragg-Brentano mode at room temperature) agreed with that calculated from a standard structure taken from International Zeolite Association Structure Database.

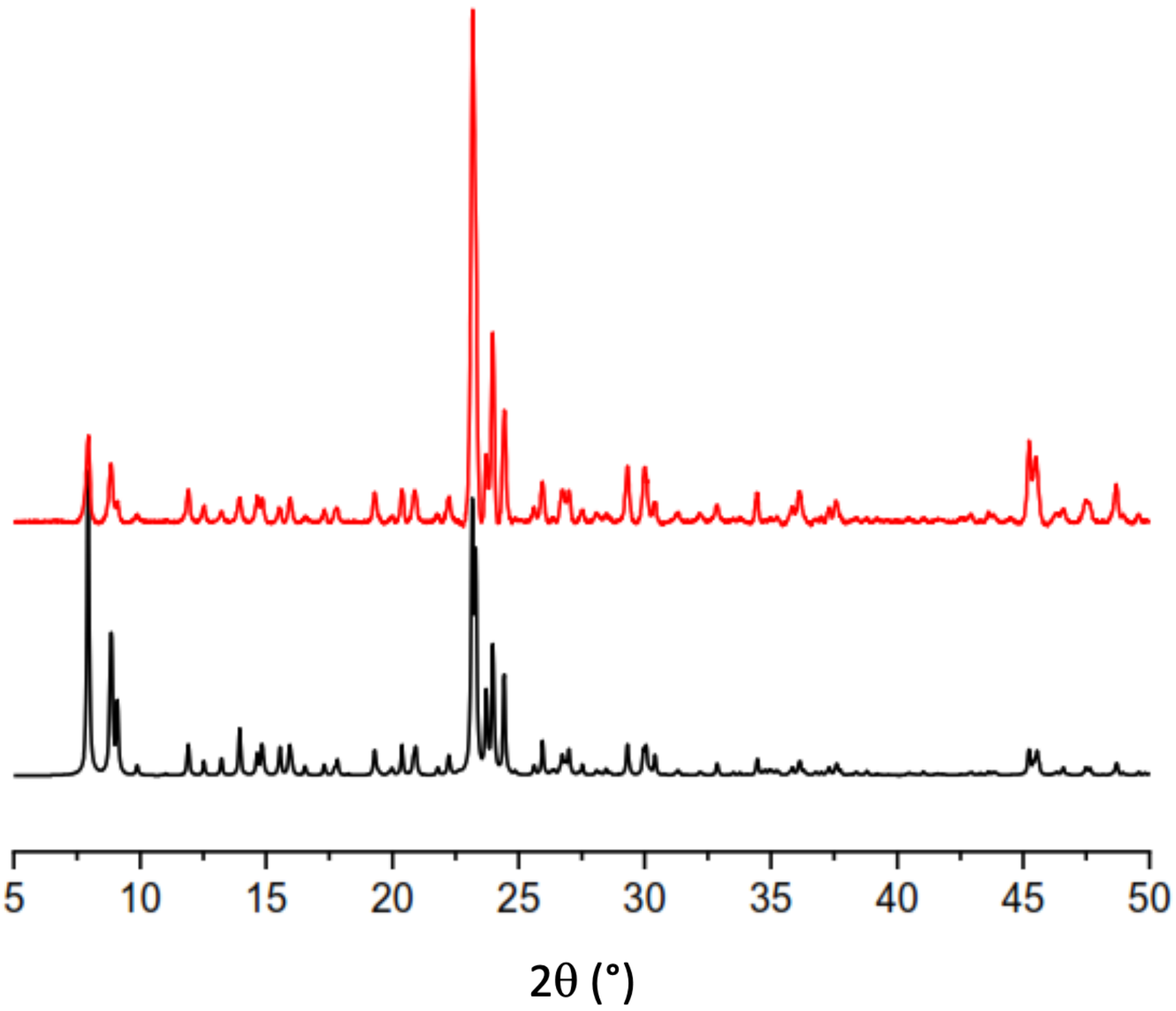

Figure S1. PXRD patterns for calcined nanosized siliceous MFI (red) and compare against a simulated pattern (black) calculated based on the structure in the International Zeolite Association (https://www.iza-online.org) Structure Database (black).

The isotope enrichment on the microporous liquid mixing 50 mg of zeolite with 0.5 mL of water enriched to 40% in ^{17}O in a small sample vial for six days. The solid MFI nanoparticles were then recovered through centrifugation. Thermogravimetric analysis (Figure S2) indicated that this sample

lost a small amount (~5% of the total mass of the sample) of weakly held, probably physisorbed water, at temperatures below 100 °C. Prior to NMR spectroscopy experiments the samples were therefore dried at 80 °C overnight in an oven to remove this extra water. The TGA also shows a small mass lost (~0.5%) between ~100 and 350 °C – this is ascribed to more strongly adsorbed water inside the pores of the zeolite as described in reference 17 in the main paper.

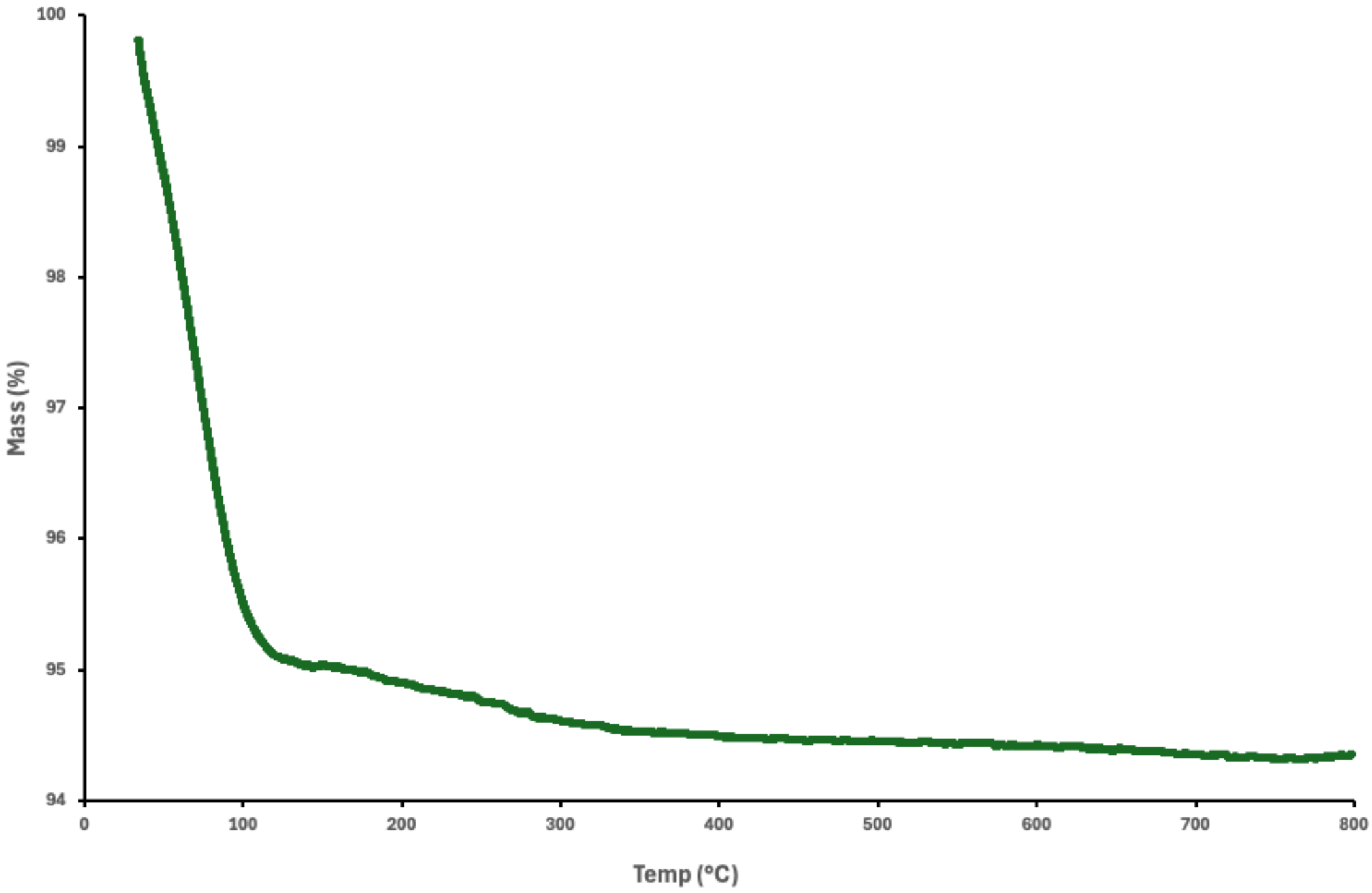

Figure S2 The TGA, shown above, shows a mass loss of ~5% of mass below 100 °C. This is usually ascribed to weakly adsorbed water primarily at the surface. There is a very small further mass loss (~0.4%) up to about 350 °C which we ascribe to a very small amount of water adsorbed in the pores.

S2 NMR spectroscopy

All ^{29}Si solid-state NMR measurements (e.g. Figure S3) were performed using a Bruker Avance III spectrometer equipped with a 9.4 T wide-bore magnet, operating at a Larmor frequency of 79.47 MHz using a Bruker 4 mm HFXY probe at a spinning frequency of 10 kHz. ^{29}Si MAS NMR spectra were acquired by signal averaging of between 112 and 496 transients with a recycle interval of 120 s and a radiofrequency nutation rate of ~83 kHz. ^{29}Si CP MAS NMR spectra were acquired by averaging 800 transients with a recycle interval of 10 s, using a ^{1}H $\pi/2$ pulse with a radiofrequency nutation rate of ~71 kHz and a 9 ms contact pulse for the transfer of magnetisation from ^{1}H to ^{29}Si. During acquisition, SPINAL-64[3] ^{1}H heteronuclear decoupling was applied with a pulse length of and radiofrequency nutation rate of ~62.5 kHz.

All ^{17}O solid-state NMR measurements were performed using a Bruker Avance III spectrometer equipped with a 14.1 T wide-bore magnet operating at a Larmor frequency of 81.34 MHz, using a Bruker 3.2 mm HX probe and a spinning frequency of 20 kHz. ^{17}O MAS NMR spectra were acquired by averaging 128 (calcined) or 1280 (as-made) transients with a recycle interval of 15 s using a $\pi/12$ short-flip angle pulse with a radiofrequency nutation rate of ~71 kHz. The ^{17}O MQMAS spectra were acquired using a z-filter[4] pulse sequence, with a radiofrequency field strength of ~71 kHz for the triple-quantum excitation and conversion pulse, while the final central-transition selective pulse had a strength of ~14 kHz. In the MQMAS spectrum 1680 transients were averaged with a recycle interval of 1 s for each of

50 t_1 increments. The MQMAS spectra are shown after a shearing transformation to allow the projection of the isotropic spectrum directly onto the indirect dimension and are referenced using the convention in Pike *et al.*[5] The ^{17}O spectra for the enriched samples were compared with a spectrum of the unenriched sample (Figure S4) to show that no signal is measured on the same time for natural abundance zeolite sample.

All ^{29}Si chemical shifts are shown in ppm relative to $Si(CH_3)_4$, using the $OSi(CH_3)_3$ resonance of oktakis(trimethylsiloxy)silsequioxane (Q_8M_8) (δ = 11.5 ppm) as a secondary reference. All ^{17}O chemical shifts are shown in ppm relative to D_2O.

In solid-state NMR spectroscopy the interactions that affect the nuclear spins have both isotropic and anisotropic (orientation dependent) terms. In solution, the rapid tumbling motion of the molecules removes anisotropic terms. For solids, this motion is mimicked by rapid magic-angle spinning (MAS); rotation of the sample around an axis inclined at an angle of 54.736° with respect to the external magnetic field. For quadrupolar nuclei (I > 1/2) NMR spectra are broadened by the second-order quadrupolar interaction which is not removed by MAS. This results in an isotropic shift of the signal, δ_Q, and an anisotropic broadening in a powdered solid. The tensor that describes the interaction can be parameterised to give a magnitude, C_Q and an asymmetry, η_Q. In some NMR experiments, it can be useful to use a composite parameter, the quadrupolar product, $P_Q = C_Q (1+ \eta_Q^2/3)$. In solid-state NMR spectra of quadrupolar nuclei, the position of any signal in the NMR spectrum, usually denoted, δ, is the sum of contributions from the isotropic chemical shift/shielding, δ_{iso}, and the quadrupolar shift, δ_Q.

Multiple-quantum (MQ) MAS is a two-dimensional NMR experiment that correlates a multiple-quantum transition with a single-quantum central transition to remove the second-order quadrupolar broadening. This yields, after the relevant processing, a two-dimensional spectrum that contains ridge like lineshapes for each distinct species present with a high-resolution isotropic spectrum (free from any broadening) obtained parallel to these. The position of the centre-of-gravity of a signal in the two-dimensional spectrum is given by line positions in the vertical (δ_1) and horizontal (δ_2) dimensions. From this position it is possible to extract the values of δ_{iso} and P_Q.

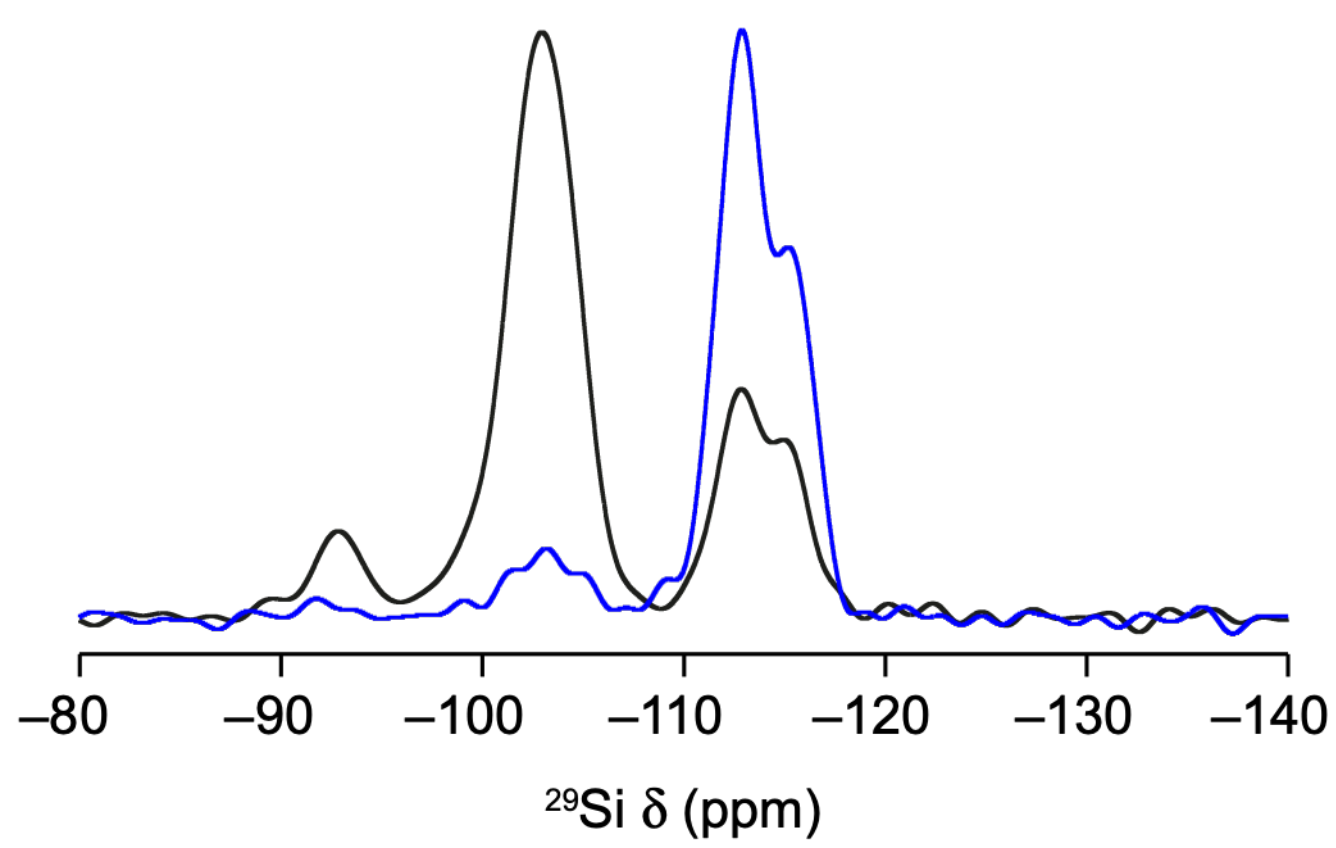

Figure S3 ^{29}Si (14.1 T, 10 kHz) MAS (blue) and CP MAS NMR (black) spectra of calcined nanosized siliceous MFI. The ^{29}Si MAS NMR spectrum shows two sets of signals corresponding (in order of decreasing chemical shift) to Q^3 and Q^4 Si atoms. The ^{1}H-^{29}Si CP MAS NMR spectrum shows three peaks, with a low-intensity Q^2 signal at higher δ, which is not clearly visible in the ^{29}Si MAS NMR spectrum.

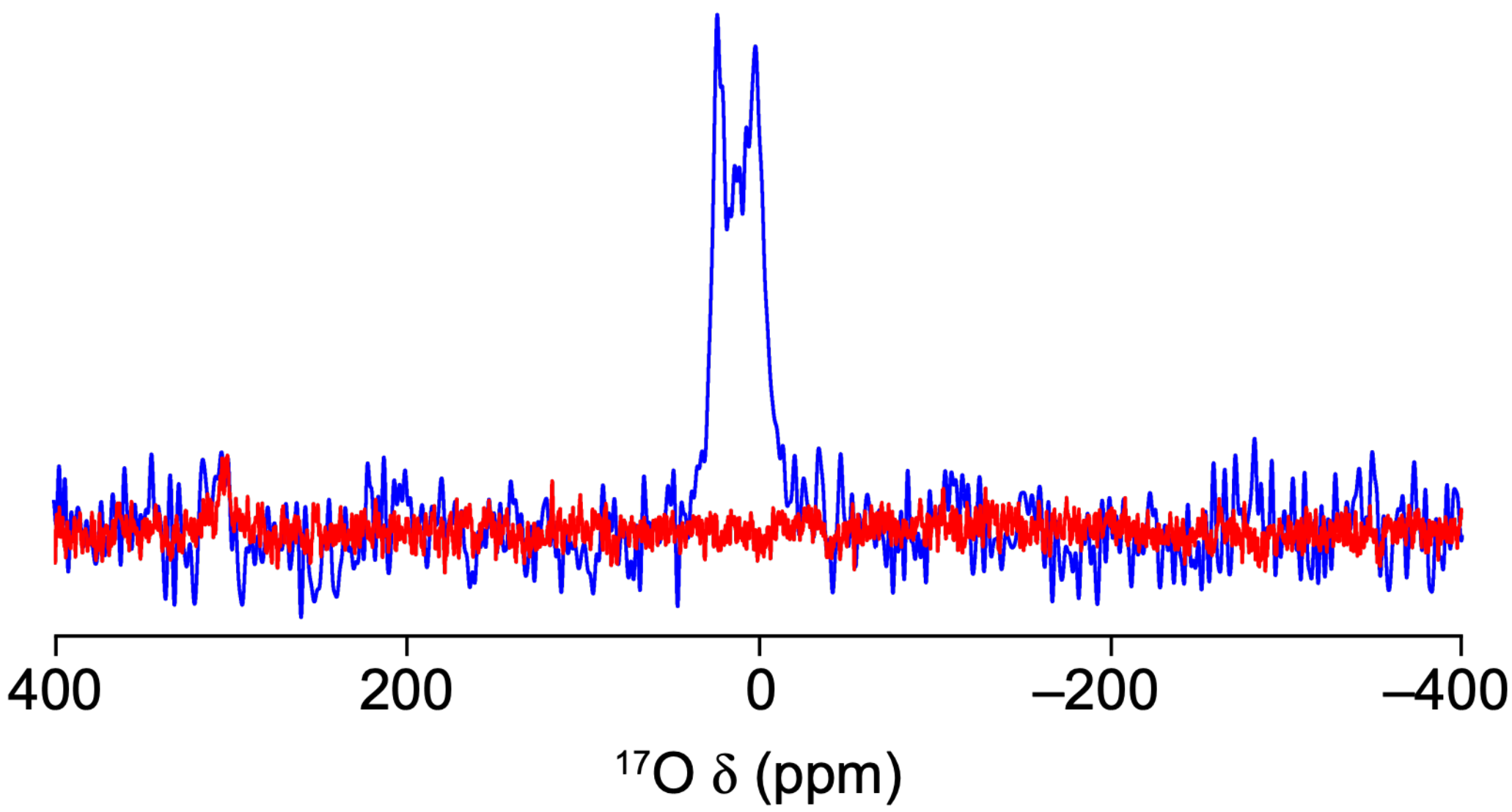

Figure S4 ^{17}O spectra (14.1 T, 20 kHz) MAS NMR spectra collected on an unenriched (natural abundance) sample of MFI nanoparticles (red) compared to the isotopically enriched sample (blue). Note the red spectrum was recorded on an Avance NEO X spectrometer. The spectra are scaled such that the very small peak resulting from the rotor (at ~360 ppm) is the same height in both.

S3 Dynamic light scattering (DLS) measurements

DLS measurements were performed on all samples to observe the changes in the hydrodynamic size of the nanoparticles in the microporous water before and after contact with enriched water. The sample preparation involved making 0.25mg L^{-1} of the siliceous MFI nanoparticles in distilled water. Firstly, the distilled water was filtered to ensure no particles were present. Then a 1 mg L^{-1} solution of siliceous MFI nanoparticles was made up by dispersing 3 mg of zeolite in 3 mL of water. Subsequently, this solution was diluted to make a 0.25 mg L^{-1} of siliceous MFI nanoparticles in distilled water for DLS

measurements. All the DLS measurements were performed on a Malvern Zetasizer Pro instrument using a 10 mm path length glass cuvette placed in a thermostatted cell holder at constant temperature of 25 °C. Six measurements were repeated for each sample.

Trial	1	2	3	4	5	6	Mean
Size / nm	59.1	68.7	59.1	50.8	79.9	59.1	**62.8 ± 10.1**

Table S1 DLS measurements for the synthesised calcined nanosized siliceous MFI sample dispersed in water. The reported hydrodynamic ratio of the nanoparticles is the mean of six different measurements.

S4 Scanning Electron Microscopy (SEM) imaging

All SEM measurements used a JEOL JSM-IT800 SEM instrument. All samples were dissolved in ethanol and added dropwise to Al foil. SEM images after recovery of the nanoparticles from the microporous water showed approximately spherical crystallites with an approximate diameter of 60-70 nm (Figure S3).

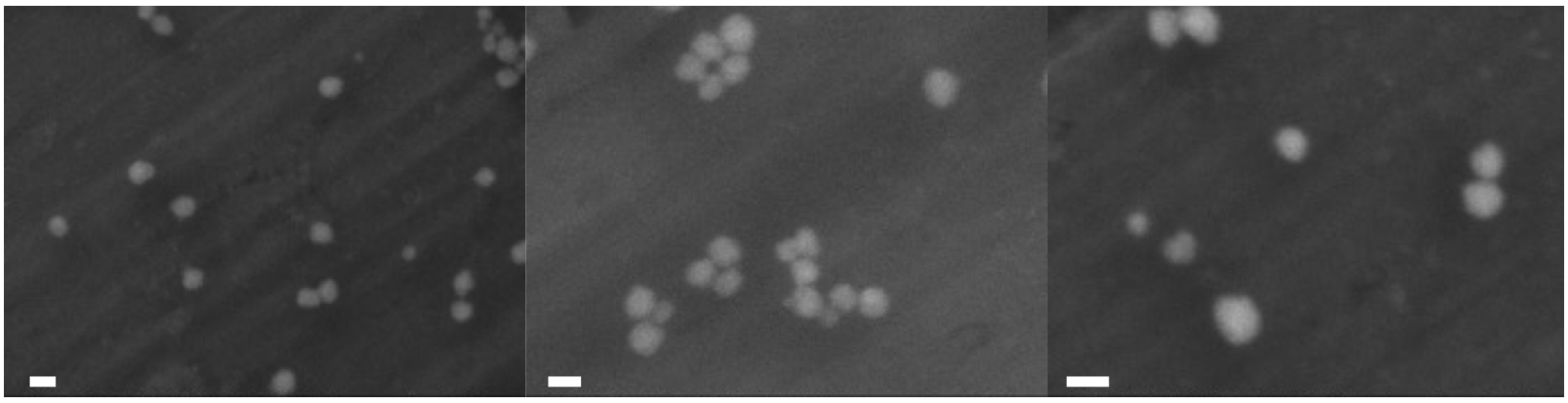

Figure S5. SEM images for the calcined nanosized siliceous MFI with magnifications 60k, 80k and 100k. All scale bars are 100 nm

S5 Transmission Electron Microscopy (TEM)

All TEM imaging was performed using a FEI Titan Themis TEM. The MFI samples were dispersed in ethanol and then added dropwise on lacey carbon/copper grids before being measured.

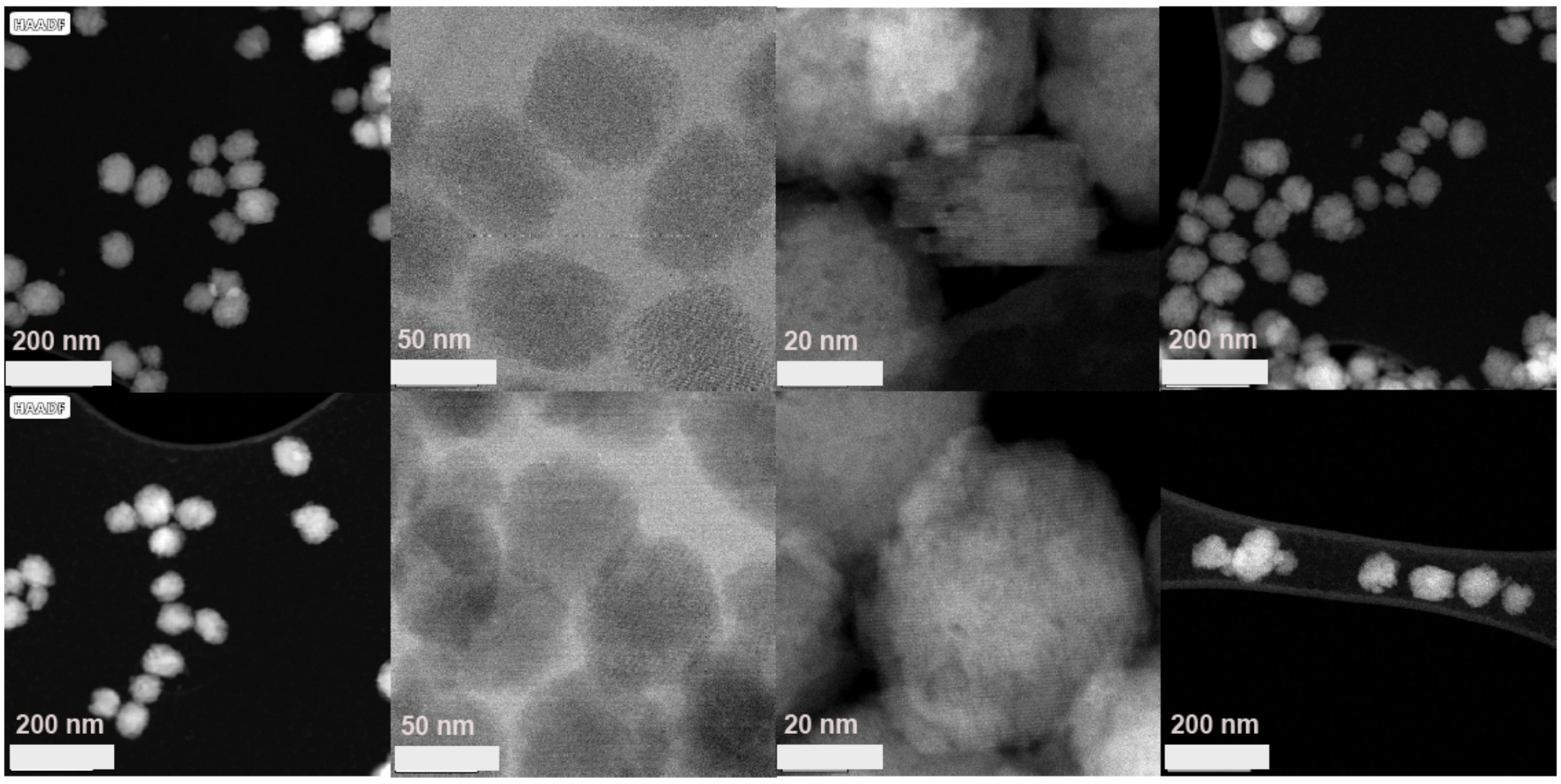

Figure S6. Various high-angle annular dark-field (HAADF) TEM images for the calcined siliceous MFI (top) and ^{17}O-enriched calcined nanosized siliceous MFI zeolite (bottom) at varying magnification.

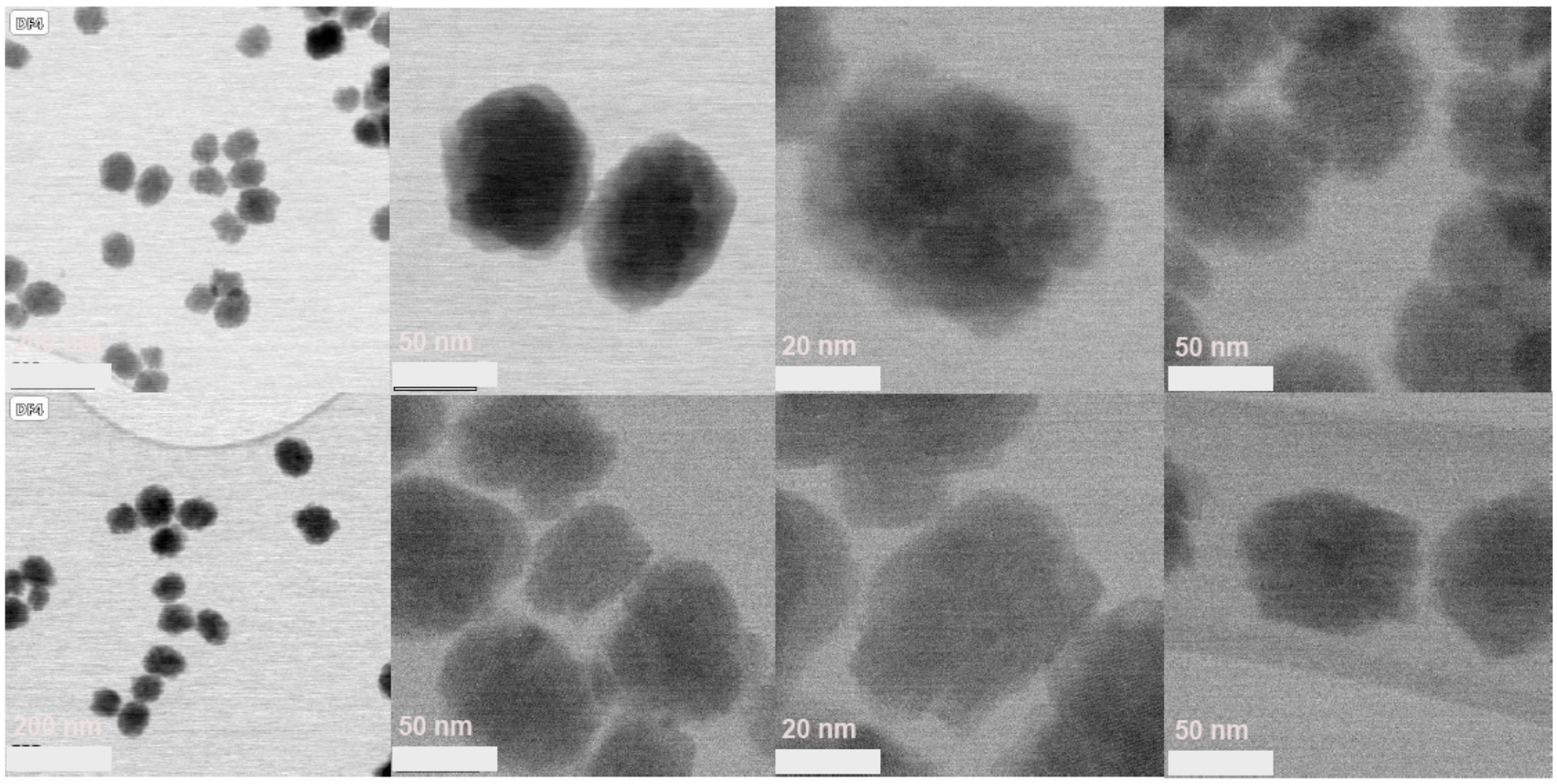

Figure S7. Various dark field (DF) TEM images for the calcined siliceous MFI (top) and ^{17}O-enriched calcined nanosized siliceous MFI zeolite (bottom). The scale bars are, from left to right, 200 nm, 50 nm, 20 nm and 50 nm).

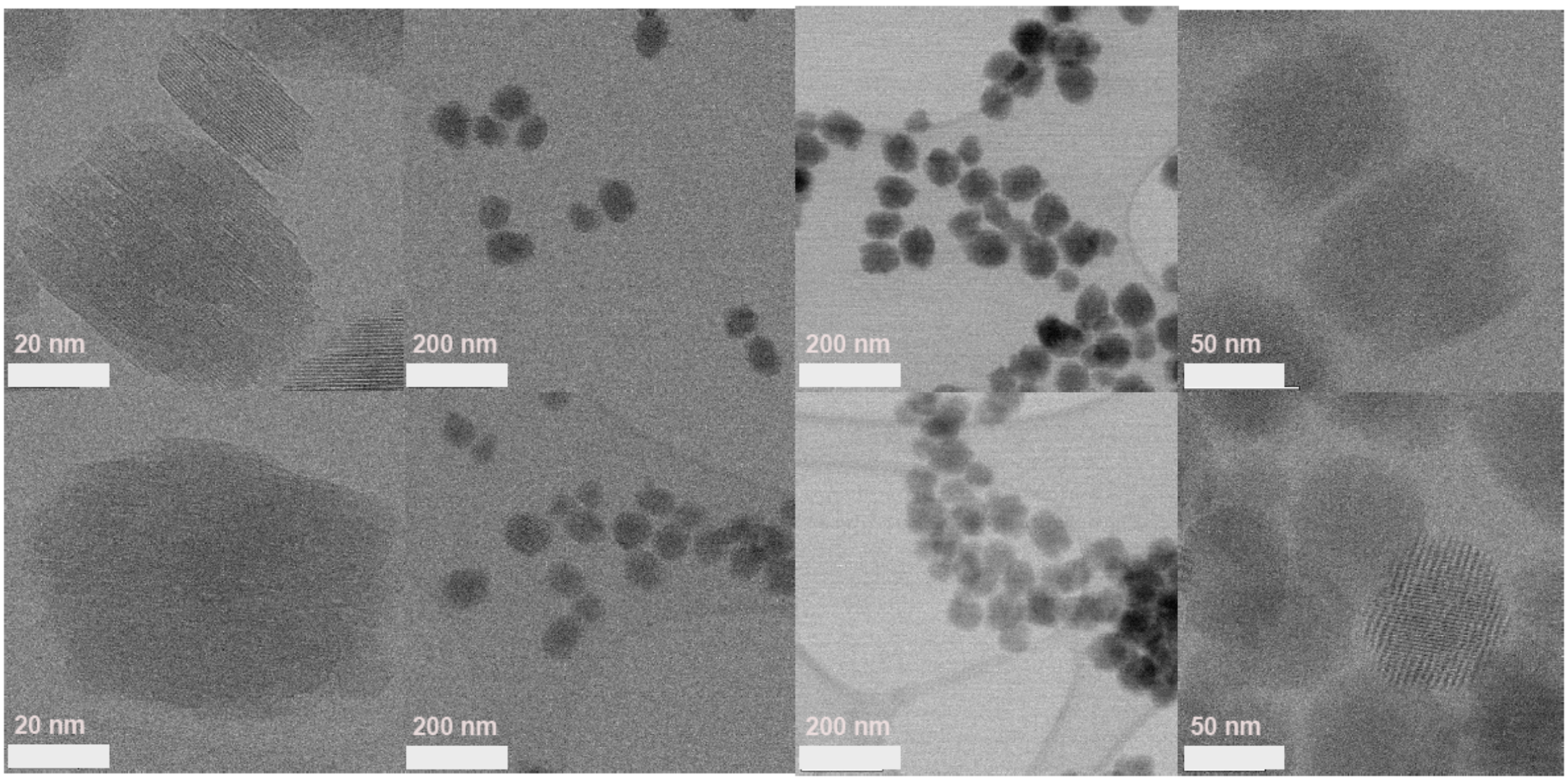

Figure S8. The bright field (BF) TEM images for the calcined siliceous MFI (top) and ^{17}O-enriched calcined nanosized siliceous MFI zeolite (bottom).

References:

1. B. J. Schoeman, J. Sterte and J. E. Otterstedt, *Zeolites*, 1994, **14**, 568–575.
2. S. Mintova, N. H. Olson, J. Senker and T. Bein, *Angew. Chem. Int. Edit.*, 2002, **41**, 2558–+.
3. B. M. Fung, A. K. Khitrin and K. Ermolaev, *J. Magn. Reson.*, 2000, **142**, 97–101.
4. J. P. Amoureux, C. Fernandez and S. Steuernagel, *J. Magn. Reson. Ser. A*, 1996, **123**, 116–118.
5. K. J. Pike, R. P. Malde, S. E. Ashbrook, J. McManus and S. Wimperis, *Solid State Nucl. Mag. Res.*, 2000, **16**, 203–215.
